# F120. USING DIGITAL MEDIA ADVERTISING IN EARLY PSYCHOSIS INTERVENTION

**DOI:** 10.1093/schbul/sby017.651

**Published:** 2018-04-01

**Authors:** Michael Birnbaum, Chantel Garrett, Amit Baumel, Asra Rizvi, Whitney Muscat, John Kane

**Affiliations:** 1 Northwell Health; 2 Strong 365 (One Mind)

## Abstract

**Background:**

Identifying and engaging youth with early stage psychotic disorders in order to facilitate timely treatment initiation remains a major public health challenge. While advertisers routinely use the internet to directly target consumers, limited efforts have focused on applying available technology to proactively encourage help seeking in the mental health community. This study explores how one might take advantage of Google Adwords in order to reach prospective patients with early psychosis.

**Methods:**

A landing page was developed with the primary goal of encouraging help seeking individuals in New York City to contact their local early psychosis intervention clinic. In order to provide the best opportunity to reach the intended audience, Google AdWords was utilized linking over 2,000 manually selected search terms to strategically placed landing page advertisements. The campaign ran for 14 weeks between April 11th and July 18th 2016 with a total budget of $1427.

**Results:**

The ads appeared 191,313 times and were clicked on 4,350 times at a per-click cost of $.33. Many users took additional help seeking steps including obtaining psychosis specific information/education (n=1,918 / 44%), completing a psychosis self-screener (n=671 / 15%) and contacting the Early Treatment Program (n=57 / 1%).

**Discussion:**

Digital ads appear to be a reasonable and cost effective method to reach individuals who are searching for behavioral health information online. More research is needed to better understand the many complex steps between online search inquiries and making first clinical contact.

